# Patient-reported outcomes after operative versus nonoperative treatment of pediatric lateral humeral condyle fractures

**DOI:** 10.1097/MD.0000000000027440

**Published:** 2021-10-15

**Authors:** Ijezie A. Ikwuezunma, Krishna V. Suresh, Derek T. Nhan, Barry R. Bryant, Ronak N. Kotian, R. Jay Lee

**Affiliations:** Department of Orthopaedic Surgery, The Johns Hopkins Hospital, 601 N. Caroline Street, Baltimore, MD.

**Keywords:** clinical outcomes, lateral humeral condyle fracture, open reduction, patient-reported outcomes, percutaneous reduction

## Abstract

Lateral humeral condyle fractures in children are treated with several approaches, yet it is unclear which has the best treatment outcomes. We hypothesized that functional outcomes would be equivalent between treatment types, reduction approaches, and fixation types. Our purpose was to assess patient-reported outcomes and complications by treatment type (operative versus nonoperative), reduction approach (open versus percutaneous), and fixation type (cannulated screws versus Kirschner wires).

We retrospectively reviewed data from acute lateral humeral condyle fractures treated at our level-1 pediatric trauma center from 2008 to 2017. Patients were included if they were 8 years or older and had completed clinical follow-up. Fractures were categorized by fracture severity as mild (<2-mm displacement), moderate (isolated, 2- to 5-mm displacement), or severe (isolated, >5-mm displacement or >2-mm displacement with concomitant elbow dislocation or other elbow fracture). We extracted data on patient age, sex, treatment type, reduction approach, fixation type, patient-reported outcomes (shortened Disabilities of the Arm, Shoulder, and Hand and Patient Reported Outcome Measurement Information System upper extremity), treatment complications, and follow-up duration. Patients in the operative versus nonoperative group and across fracture severity subgroups did not differ significantly by age, sex, or follow-up duration. Bivariate analysis was performed to determine whether outcomes differed by intervention. Alpha = 0.05.

No differences were observed in patient-reported outcomes between operative versus nonoperative groups for the mild and severe fracture subgroups. No differences were observed between approach (open versus percutaneous) or instrumentation (cannulated screw versus Kirschner wire fixation) for any outcome measure within the operative group. Patients whose fractures were stabilized with screws versus wires had significantly higher rates of return to the operating room (94% versus 8.3%, *P* < .001). The overall complication rate for our cohort was low, with no differences by treatment type or fracture severity.

In our cohort, patient-reported outcomes were similar across fracture severity categories, irrespective of treatment or fixation type. Patients who underwent internal fixation with cannulated screws experienced significantly higher rates of return to the operating room compared with those treated with Kirschner wires but otherwise had similar complication rates and patient-reported outcomes.

**Level of Evidence:** 3

## 1 Introduction

Fractures of the lateral humeral condyle are common in children, accounting for approximately 20% of all distal humeral fractures, and usually result from a direct force of the radial head on the condyle or avulsion forces form the lateral ligament.^[1]^ Several complications are associated with pediatric lateral humeral condyle fractures, including malunion or nonunion, angular deformity, avascular necrosis, ulnar nerve palsy, and physeal arrest.^[2,3]^

To date, the treatment choice for lateral humeral condyle fractures remains controversial. Whereas nonoperative management with cast immobilization is preferred for fractures displaced less than 2 mm, surgical treatment is used for fractures with moderate to severe displacement (greater than 2 mm). Evidence, however, suggests the need for flexibility in these guidelines.^[3–6]^ Absolute indications for operative treatment are open fracture and nonreducible fracture. However, indication for operative treatment also depends on the degree of displacement of the lateral condyle fracture fragment. Studies indicate that nondisplaced, stable fractures (typically defined as involving <2 mm of displacement) can be treated with cast immobilization with close follow-up.^[7]^ Although operative treatment is typically recommended for displacement of >2 to 3 mm, the studies supporting this recommendation have been retrospective, lack comparison groups, and assess only radiographic healing without accounting for cosmetic and functional outcomes.^[8–10]^

For patients treated operatively, it is unclear whether internal fixation with Kirschner wires (K-wires) or cannulated screws has superior patient outcomes. Studies have reported similar union rates and low complication rates with both methods.^[3,11]^ In a retrospective review of 62 children, Li and Xu^[12]^ found no difference in clinical outcomes for fractures stabilized with K-wires or screws but reported that K-wires required a longer period of fixation and local skin care, whereas screws presented a risk of prominence and typically required a second surgical procedure for removal to prevent growth disturbance. Although both methods produced sufficient repair as assessed radiographically, as well as low complication rates, a study of patient-reported functional outcomes (eg, the ability to perform daily tasks) has not yet been performed.

The purpose of this study was to assess patient-reported outcomes and complications by treatment type (operative versus nonoperative), reduction approach (open versus percutaneous), and fixation type (cannulated screws versus K-wires) to test the hypothesis that functional outcomes would be equivalent between treatment types, reduction approaches, and fixation types.

## 2 Methods

This study was approved by our institutional review board. Parental verbal consent was obtained before survey administration. The article complies with STROBE (Strengthening the Reporting of Observational Studies in Epidemiology) guidelines.

### 2.1 Patient selection

Using a database of patients treated by the orthopaedic surgery department, we retrospectively reviewed the medical records of all children (<18 years old) with acute fractures of the lateral humeral condyle who presented to our US level-1 pediatric trauma center between January 2008 and June 2017. We included patients aged 8 to 17 years at follow-up (the range for which the patient-reported outcome measures are validated) who had available preoperative or postoperative radiographs and who had complete clinical follow-up (for valid completion of the patient-reported outcome measures). We excluded patients who lacked clinical follow-up, who were unable to be contacted by telephone, or whose parents declined participation. We extracted the following data from patients’ medical records: age at time of fracture, sex, height, and weight, side of the fracture, treatment method (operative or nonoperative), surgical approach (open or percutaneous), type of fixation (K-wires or screws), and treatment complications.

### 2.2 Patient cohort

In total, 116 children (26 girls) with a mean (± standard deviation) age of 6.4 ± 2.9 years at the time of fracture were treated for lateral humeral condyle fractures during the study period. The operative and nonoperative groups for each fracture severity subgroup were not significantly different with respect to age (except among those with severely displaced fractures), sex, side of the fracture, and duration of follow-up (Table 1). There were also no differences in mean follow-up duration according to whether the procedure was performed using an open approach or percutaneously or according to the type of fixation used.

**Table 1:** Patient and injury characteristics by fracture severity and treatment type for 116 pediatric patients with lateral humeral condyle fractures.

	Mild fracture (N = 41)					Moderate fracture (N = 29)					Severe fracture (N = 46)				
	Operative (N = 7)		Nonoperative (N = 34)			Operative (N = 22)		Nonoperative (N = 7)			Operative (N = 43)		Nonoperative (N = 3)		
Characteristic	Mean ± SD	N (%)	Mean ± SD	N (%)	*P*	Mean ± SD	N (%)	Mean ± SD	N (%)	*P*	Mean ± SD	N (%)	Mean ± SD	N (%)	*P*
Age (yr)	5.6 ± 3.0		6.2 ± 2.4		.52	6.5 ± 2.3		8.1 ± 5.8		.26	6.1 ± 2.8		9.7 ± 1.5		.04
Male sex		5 (71)		29 (85)	.38		14 (64)		6 (86)	.27		34 (79)		2 (67)	.62
Right elbow		5 (71)		19 (56)	.45		9 (41)		2 (29)	.56		16 (37)		0 (0)	.19
Follow-up (d)	84 ± 19^∗^		79 ± 17^†^		.90	127 ± 104		81 ± 43		.27	227 ± 291		69 ± 39		.36

### 2.3 Fracture severity

We categorized the 116 patients who met our inclusion criteria by fracture severity according to the Jakob and Song classification systems, as follows: mild (<2-mm displacement), moderate (isolated, 2- to 5-mm displacement), or severe (isolated, >5-mm displacement or >2-mm displacement with concomitant elbow dislocation or other elbow fracture).^[13,14]^ Fracture displacement was measured on initial radiographs using Carestream Vue PACS (Carestream Health, Inc., Rochester, NY) by a pediatric orthopaedic surgeon. Maximum displacement on any radiographic view was recorded.

### 2.4 Treatment

Treatment decisions were guided by the preferences of the 4 fellowship-trained pediatric orthopaedic surgeons. Seventy-two patients underwent operative treatment, and 44 patients underwent nonoperative treatment (Table 2). Nonoperative treatment consisted of long-arm cast immobilization for 4 to 8 weeks, dependent on radiographic evidence of healing. Operative treatment consisted of open or percutaneous fixation using K-wires or partially threaded cannulated screws by 1 of 4 fellowship-trained pediatric orthopaedic surgeons, followed by cast immobilization. Treatment methods and timing of immobilization varied slightly among each surgery.

**Table 2:** Procedure type by fracture severity for 72 pediatric patients with operatively treated lateral humeral condyle fractures.

Procedure Type	N (%)		
	Mild (N = 7)	Moderate (N = 22)	Severe (N = 43)
Percutaneous fixation	6 (86)	12 (55)	17 (40)
Open fixation	1 (14)	10 (45)	26 (60)
Cannulated screws	5 (71)	13 (59)	21 (49)
Kirschner wires	2 (29)	9 (41)	22 (51)

### 2.5 Complications

Radiographs taken during subsequent follow-up visits were reviewed for signs of nonunion or delayed healing, which was defined as no evidence of fracture callus or bony union at 6 weeks or more after treatment. Rates of return to the operating room, including for instrumentation removal, were also collected.

### 2.6 Patient-reported outcomes survey

We attempted to contact all 116 eligible patients by telephone to ask them to complete the outcome measure questionnaires. Informed consent was obtained from all parents and guardians, and assent was obtained from all patients younger than 18 years. Patients were asked to complete the shortened Disabilities of the Arm, Shoulder, and Hand (QuickDASH) and Patient Reported Outcome Measurement Information System (PROMIS) Upper Extremity questionnaires. If the child was unavailable, proxy forms of the surveys were administered to parents/guardians by telephone. Patients or their proxies were also asked how pleased they were on a 10-point scale (with 0 being not pleased at all and 10 being completely satisfied) with the appearance of the extremity and the care they received. Of 116 patients, 44 patients (38%; 5 girls; mean age 6.8 ± 2.9 years at the time of fracture) completed PROMIS and QuickDASH surveys. We were unable to contact the remaining 72 patients.

Among the patients who completed patient-reported outcome measures, mean duration of follow-up was 4.4 ± 2.6 years. Those who completed the QuickDASH and PROMIS surveys also had no significant differences in age, duration of follow-up, laterality, or sex between nonoperative and operative treatment groups across all fracture severities, with the exception of sex among those with moderately displaced fractures. When stratifying by fracture severity, there were no differences in mean follow-up duration according to whether the procedure was performed using an open or percutaneous approach or according to the type of implant used.

### 2.7 Statistical analysis

Data were analyzed using Stata, version 15.0, software (StataCorp LLC, College Station, TX). Descriptive statistics were calculated for all data collected. Categorical variables are expressed as counts with percentages. Categorical outcomes were analyzed using Fisher exact tests. Continuous variables are reported as means with standard deviations. Continuous variables were analyzed using Student *t* tests for normally distributed data and Mann–Whitney *U* tests for non-normally distributed data. Shapiro–Wilk tests were used to test for normal distribution of continuous data. Two-tailed *P*-values <.05 were considered significant.

## 3 Results

### 3.1 Patient-reported outcomes

No significant differences were observed in mean QuickDASH scores or normalized PROMIS scores between operative versus nonoperative treatment for the mild and severely displaced fracture groups (Table 3). Mean appearance scores between patients treated operatively versus nonoperatively did not differ significantly by severity group. In the operative cohort, we found no difference in mean patient-reported outcome scores between open versus percutaneous reduction (for QuickDASH, open group = 11, percutaneous group = 13, *P* = .08; for PROMIS, open group = 30, percutaneous group = 29, *P* = .16). Likewise, no difference in mean patient-reported outcome scores was observed between treatment with cannulated screws versus K-wires (for QuickDASH, both groups = 12, *P* = .68; for PROMIS, both groups = 30, *P* = .34).

**Table 3:** Mean (standard deviation) patient-reported outcome scores by fracture severity and treatment type for 44 pediatric patients with lateral humeral condyle fractures.

Measure	Mild (n = 13)			Moderate (n = 6)			Severe (n = 25)		
	Operative (n = 2)	Nonoperative (n = 11)	*P*	Operative (n = 5)	Nonoperative (n = 1)	*P*	Operative (n = 22)	Nonoperative (n = 3)	*P*
Normalized QuickDASH	13 (2.1)	11 (0.9)	.17	12 (0.89)	12 (NA)	NA	12 (2.9)	11 (0)	.51
Normalized PROMIS UE	28 (3.5)	30 (1.8)	.24	30 (0.45)	30 (NA)	NA	30 (0.64)	30 (0)	.72
Pleased with care	10 (0)	9.9 (0.3)	.69	9.6 (0.89)	10 (NA)	NA	9.1 (1.5)	10 (0)	.31
Pleased with appearance	8.5 (0.71)	9.7 (0.9)	.10	9.2 (1.8)	10 (NA)	NA	8.4 (1.8)	7.8 (2.0)	.62

### 3.2 Complications

Among the 116 patients in this study, 2.7% (2/72) operatively treated patients and 4.5% (2/44) nonoperatively treated patients experienced delayed bone healing. All other fractures achieved union by 13 weeks after the injury with continued observation. Among the entire cohort, patients treated with screws experienced a significantly higher rate of return to the operating room (90%) than those treated with K-wires (9.1%) (*P* < .001). The findings are summarized in Table 4.

**Table 4:** Timeline for diagnosis and treatment of nonunion by operatively versus nonoperatively treated patients.

Patients	No. of Weeks		
	From index injury to nonunion diagnosis	From nonunion diagnosis to treatment	Nonunion treatment
Operatively treated			
Patient 1	15	15	Open reduction and screw fixation
Patient 2	15	1	Kirschner-wire fixation
Nonoperatively treated			
Patient 1	5.5	0.3	Closed reduction
Patient 2	12.8	3	Closed reduction

## 4 Discussion

The main findings of this study were that no differences were observed in patient-reported outcomes between operative versus nonoperative groups for the mild and severe fracture subgroups. Furthermore, no differences were observed between surgical approaches (open versus percutaneous) or instrumentation (cannulated screw versus K-wire fixation) for any outcome measure within the operative group. Patients whose fractures were stabilized with screws had significantly higher rates of reoperation compared with patients treated with K-wires. The overall complication rate for our cohort was low, with no differences by treatment type or fracture severity. These findings are relevant for daily clinical practice because they indicate that orthopaedic surgeons can safely and conveniently use their method of choice for treatment of lateral humeral condyle fractures.

Recommendations for the treatment of lateral condyle fractures have been based largely on the degree of displacement, as well as expert opinion and surgeon preference given the lack of high-quality evidence.^[15]^ This study expands on the limited evidence regarding patient-reported outcomes after pediatric lateral condyle fractures of the humerus and suggests that both nonoperative and various operative approaches are valid options. No significant differences were found in patient-reported function between patients treated operatively versus nonoperatively, open versus percutaneously, or between patients whose fractures were stabilized with K-wires versus screws.

In orthopaedic practice and research, the PROMIS measure has gained popularity for its broad coverage of health domains, relative reliability, and reduced respondent burden.^[16]^ Overbeek et al^[17]^ further established the correlation between PROMIS and the previously validated QuickDASH questionnaires as disability measures of the upper extremity. Yet to date, very few studies comparing outcomes in pediatric patients presenting with humeral fractures have focused on patient-reported outcome measures. In our series we found no significant difference in mean PROMIS or QuickDASH scores among pediatric patients treated either operatively or nonoperatively for lateral condyle humeral fractures, regardless of degree of displacement. That patient-reported outcome scores were all within a range of acceptable limits may also have been expected. Studies have shown excellent functional outcomes after lateral condyle humeral fractures, irrespective of treatment choice.^[18]^ We believe ours is the first study to report how outcomes of different reduction techniques across a spectrum of increasing displacement compare from the patient's perspective.

Previous studies are unclear as to whether minimally displaced fractures require operative treatment given the intra-articular nature of the fracture and the increased risks of delayed union, malunion, disturbed growth and avascular necrosis associated with nonoperative treatment.^[19]^ Bast et al^[20]^ showed in a cohort of 95 children with fracture displacement of <2 mm, that only 2 patients required subsequent operative treatment for further displacement. Pirker et al^[21]^ similarly showed in a group of 51 patients that cast immobilization was sufficient to achieve union of minimally displaced fractures with relatively low risk for eventual surgery. Greenhill et al^[22]^ reported that patients treated nonoperatively had significantly fewer clinic visits, less x-ray exposure, and a clinically negligible increase in average maximum displacement at final follow-up compared with their peers whose fractures were stabilized with in situ pinning. However, others have suggested that these fractures should be reduced surgically to lower the risk of delayed displacement after nonoperative treatment, resulting in malunion, nonunion, and angular deformities.^[6,23,24]^ Though our ability to make definitive recommendations is limited by small subgroup sizes, our study does report on the largest comparative cohort assessed using validated outcome measures with a nearly even distribution of patients across fracture severity categories. Our report provides evidence that isolated lateral condyle fractures with mild or severe displacement greater than 5 mm can be managed effectively with either operative or nonoperative treatment.

Furthermore, among operative fractures, we found no significant differences in complication rates between patients whose fractures were stabilized operatively or not, irrespective of fracture severity. Closed reduction and percutaneous pinning has traditionally been preferred for stable fractures that can be anatomically reduced because of their small diameter and low risk of physeal disruption, though such treatment is associated with risk of infection.^[15]^ Our findings corroborate those of Li and Xu,^[12]^ who also reported greater incidence of superficial skin infection among 62 children with lateral humeral condyle fractures treated percutaneously with either K-wires (17%) or screws (0%). Additionally, Stein et al^[25]^ and Gilbert et al^[26]^ demonstrated significantly faster times to union, lower rates of open reduction, and lower infection rates with screws than with K-wires. However, although open reduction is favorable because lag screws achieve metaphyseal compression, our study demonstrates that the rate of return to the operating room was significantly greater in the screw fixation cohort.^[12,19]^ This was attributable in large part to the need for routine removal of screws to avoid growth disturbance, coupled with the need for repeat clinical evaluation. Further study is needed to clarify whether 1 treatment method is truly superior to the other with regard to both clinical and patient-reported outcomes.

Limitations of our study include the small number of patients who completed the functional outcome surveys, which prevented us from assessing patient-reported outcomes among patients with moderately displaced fractures. However, our response rate is similar to that of other studies, and though our subgroup analyses do not definitively support nonoperative versus operative treatment and K-wire versus screw fixation, our study does provide support for surgeon or patient preference when it comes to the treatment of lateral humeral condyle fractures. Another limitation relates to the imprecise methods for classifying fracture displacement. Knutsen et al^[27]^ showed poor reliability in the measurement of displacement of lateral humeral condyle fractures when comparing true values from a cadaveric arm to standard radiographic measurements. However, the use of all available views to determine maximal displacement minimizes the numbers of moderately and severely displaced fractures incorrectly assigned to our mild fracture group. Additionally, our study was limited by relatively short radiographic follow-up. However, our goal was to determine the differences in patient-reported functional outcomes, and it is likely that any short-term complications identified radiographically would be reflected in the longer-term patient survey results. Finally, though PROMIS scores have become an increasingly popular metric to assess patient-reported outcomes, our inability to detect a difference in any PROMIS domains across all of our comparisons may suggest a ceiling effect at the highest scores, which supports concerns about the ability of PROMIS to discriminate at high levels of function. Using PROMIS scores from 5202 patients, Beleckas et al^[28]^ showed the PROMIS Upper Extremity computer-adaptive test had a ceiling effect at only 0.6 standard deviations above the proposed normal population mean, thus suggesting the need for new metrics when studying the upper extremity.

In conclusion, neither nonoperative nor operative treatment with K-wire or screw fixation was associated with a difference in functional patient-reported outcomes or major complication rates among pediatric patients presenting with lateral humeral condyle fracture. Surgeons can continue to use their preference when choosing among the treatment options for lateral humeral condyle fractures. Given the limited differences in outcomes among cohorts undergoing the various operative techniques and nonoperative treatment, we believe our study encourages shared decision making between patients and health care professionals when managing lateral humeral condyle fractures.

## Acknowledgments

For their editorial assistance, we thank Jenni Weems, MS, Kerry Kennedy, BA, and Rachel Box, MS, in the Editorial Services group of The Johns Hopkins Department of Orthopaedic Surgery. We also thank the biostatistics consulting service at the Institute for Clinical and Translational Research at The Johns Hopkins University for their guidance and support.

## Author contributions

**Conceptualization:** Derek Nhan, R. Jay Lee.

**Investigation:** Ijezie Ikwuezunma, Krishna Suresh, Derek Nhan, Barry Bryant, Ronak Kotian, R. Jay Lee.

**Methodology:** Ijezie Ikwuezunma, Krishna Suresh, Derek Nhan, R. Jay Lee.

**Project administration:** R. Jay Lee.

**Supervision:** R. Jay Lee.
